# Hair cell-type dependent expression of basolateral ion channels shapes response dynamics in the frog utricle

**DOI:** 10.3389/fncel.2015.00338

**Published:** 2015-09-07

**Authors:** Alessandro Venturino, Adriano Oda, Paola Perin

**Affiliations:** Department of Brain and Behavioral Sciences, University of PaviaPavia, Italy

**Keywords:** hair cell, utricle, vestibular, neuron, transduction

## Abstract

The dynamics of vestibular afferent responses are thought to be strongly influenced by presynaptic properties. In this paper, by performing whole-cell perforated-patch experiments in the frog utricle, we characterized voltage-dependent currents and voltage responses to current steps and 0.3–100 Hz sinusoids. Current expression and voltage responses are strongly related to hair cell type. In particular, voltage responses of extrastriolar type eB (low pass, −3 dB corner at 52.5 ± 12.8 Hz) and striolar type F cells (resonant, tuned at 60 ± 46 Hz) agree with the dynamics (tonic and phasic, respectively) of the afferent fibers they contact. On the other hand, hair cell release (measured with single-sine membrane ΔCm measurements) was linearly related to Ca in both cell types, and therefore did not appear to contribute to dynamics differences. As a tool for quantifying the relative contribution of basolateral currents and other presynaptic factors to afferent dynamics, the recorded current, voltage and release data were used to build a NEURON model of the average extrastriolar type eB and striolar type F hair cell. The model contained all recorded conductances, a basic mechanosensitive hair bundle and a ribbon synapse sustained by stochastic voltage-dependent Ca channels, and could reproduce the recorded hair cell voltage responses. Simulated release obtained from eB-type and F-type models display significant differences in dynamics, supporting the idea that basolateral currents are able to contribute to afferent dynamics; however, release in type eB and F cell models does not reproduce tonic and phasic dynamics, mainly because of an excessive phase lag present in both cell types. This suggests the presence in vestibular hair cells of an additional, phase-advancing mechanism, in cascade with voltage modulation.

## Introduction

Vestibular afferent responses to head movements are characterized by a *response dynamics*, reported in terms of *gain* and *phase* of the first harmonic of afferent modulation relative to a sinusoidal motion stimulus. In vestibular organs, response dynamics (together with other features such as resting discharge and efferent modulation) are much better characterized at the postsynaptic side (Highstein et al., 2004; Eatock et al., 2006; Goldberg and Holt, 2013 and citations therein), than at the level of the corresponding presynaptic mechanisms. Coupled pre- and postsynaptic recording in the rat saccule showed that mechanical, electrical and release properties of type I hair cells significantly influence afferent dynamics (Songer and Eatock, 2013). On the other hand, in the turtle crista, although postsynaptic recordings suggest that afferent response dynamics are determined presynaptically (Goldberg and Holt, 2013), patch clamp recordings suggest that, at vestibular frequencies, dynamics are not significantly affected by hair cell basolateral currents, because hair cell responses approach passive ones for slow stimuli (Goldberg and Brichta, 2002). Similarly, in the toadfish canal, presynaptic dynamics has been almost completely linked to active hair bundle motion (Rabbitt et al., 2010), whereas the effect of basolateral currents appears minor (Rabbitt et al., 2005).

In the present study we show that, in hair cells from the frog utricle, voltage modulation by basolateral ion channels significantly affects postsynaptic dynamics at vestibular frequencies, but is not sufficient to explain postsynaptic dynamics. We chose to study the frog utricle because its hair cells (which are all type II) are morphologically and electrically similar to the well characterized frog saccular hair cells, but their output is vestibular, whereas the frog saccule is optimized for auditory-like (seismic) signals (Smotherman and Narins, 2000). Moreover, since basolateral currents from the frog crista are well characterized, studying the utricle allows functional comparisons between otolithic and canal hair cells in the same animal. The frog utricle contains gravity and vibratory afferents (Koyama et al., 1982), and afferent response has been correlated with the type of contacted hair cells. Gravity units are further divided in static (measuring linear acceleration), dynamic (measuring changes in linear acceleration), and static-dynamic (measuring both parameters). Extrastriolar (type B) hair cells have been associated to static gravity, and striolar hair cells (especially types C and F) to dynamic gravity; vibratory units are contacted by type E cells only (Baird, 1994a). For the present work we focused on extrastriolar type B and striolar type F cells.

Our results show that in hair cells from the frog utricle, voltage modulation by basolateral ion channels correlates with postsynaptic dynamics. A hair cell model with realistic ion channels reproduces the dynamics of voltage responses (low-pass gain and moderate phase lags for extrastriolar B cells, and frequency-dependent gain increase and small phase leads for striolar F cells); however, simulated quantal discharge sustained by single stochastic Ca channels does not reproduce postsynaptic dynamic features. Further refinements of the model will explore the interaction between hair bundle mechanical behavior (Rabbitt et al., 2010) and basolateral membrane electrical behavior (Farris et al., 2006; Ramunno-Johnson et al., 2010; Neiman et al., 2011), and more detailed release properties, since Ca-dynamics (Lelli et al., 2003; Castellano-Muñoz and Ricci, 2014; Magistretti et al., 2015) and ribbon synapse properties (Schnee et al., 2005; Rutherford and Roberts, 2006) can impart additional time structures on hair cell output.

## Materials and methods

### Dissection and isolation of hair cells or *in situ* preparations

Animal experiments described in this paper conformed with the rules established by the Animal Welfare Commitee of the University of Pavia for the use of animals in experimental studies, in compliance with the guidelines of the Italian Ministry of Health, the national laws on animal research, and the EU guidelines on animal research. For dissection, frogs (Rana esculenta L.) were anesthetized with MS-222, and the head was removed and pinned to a dissection chamber filled with Ringer solution (see below). The excised sensory epithelia were then cut and transferred to low-Ca solution, to undergo dissociation, treated in low-Ca for 20′ to remove the otolithic membrane and mounted in the recording dish for whole-mounts. Since K^+^ currents are sensitive to proteolytic damage by some enzymes used in cell isolation, we employed trypsin as a gentler dissociating agent as in Holt et al. (2001) or recorded currents from a few *in situ* preparations (Baird, 1994a; Armstrong and Roberts, 1998). Pharmacological experiments were usually performed on dissociated cells to get a better access to the cell surface.

## Solutions and drugs

The extracellular solutions used are summarized below (concentrations are in mM):

**Low-Ca dissociation:** NaCl 135, KCl 2.5, CaCl_2_ 0.8, MgCl_2_ 5.0, HEPES 5.0, EGTA 2.0, glucose 3.0, pyruvic acid 5, Na-ascorbate 1 (pH 7.25); **Ringer**: NaCl 126, KCl 3, CaCl_2_ 1.8, MgCl_2_ 1, HEPES 10, glucose 6 (pH 7.25); **RLC**: NaCl 128, KCl 3, MgCl_2_ 5.4, HEPES 10, glucose 6 (pH 7.25); **Cs-Internal**: CsCl 75, Cs_2_SO_4_ 30, MgCl_2_ 2, glucose 6, HEPES 10 (pH 7.25); **K-Internal**: KCl 75, K_2_SO_4_ 30, MgCl_2_ 2, glucose 6, HEPES 10 (pH 7.25).

A stock solution of TTX (50 μM in distilled water), iberiotoxin (500 μM in distilled water), CdCl_2_(100 mM in distilled water), capsaicin (50 mM in DMSO), and nimodipine (10 mM in DMSO) was made and aliquots of the stocks were added daily to extracellular solutions. All substances were from Sigma except for nimodipine (Alexis), iberiotoxin and TTX (Alomone Labs, Jerusalem).

### Patch-clamp recordings

Patch-clamp currents were recorded at room temperature (20°C) in the perforated patch variant of the whole-cell mode (Horn and Marty, 1988), using the setup and conditions described in Perin et al. (2001) or the same setup employing the Cairn Optopatch (Faversham, UK) amplifier. Currents were digitized at sampling rates from 1 to 100 kHz, filtered on-line at 1–20 kHz, and subsequently filtered off-line when needed. Amphotericin B (Sigma), which was employed for membrane perforation, was dissolved in DMSO (Sigma) and added to the internal solutions (final concentration: 0.4 mg/ml). Perforated patch experiments were performed in yellow lighting to avoid amphotericin B degradation. After complete perforation was achieved, capacitive currents were reduced by analog circuitry and Rs was actively compensated as much as possible. Final Rs values ranged between 2 and 20 MOhm. Cells where the maximal voltage drop due to Rs was larger than 5 mV or where Rs changed suddenly were discarded. Voltage protocols were corrected for calculated junction potentials. Stimulation, acquisition and data analysis were performed using pClamp software (Axon Instruments), Microsoft Excel®, Microcal Origin (OriginLab, Northampton, USA), and MATLAB (The MathWorks, Inc., USA).

Input resistance (R_i_) was calculated from the currents measured in response to ±5 mV voltage steps from Vz. Activation curves for voltage-dependent currents were obtained by fixed-point tail measurement after steps long enough to maximally activate the current of interest but not to elicit a significant inactivation (see Masetto et al., 2000 for details).

**Figure 1:**
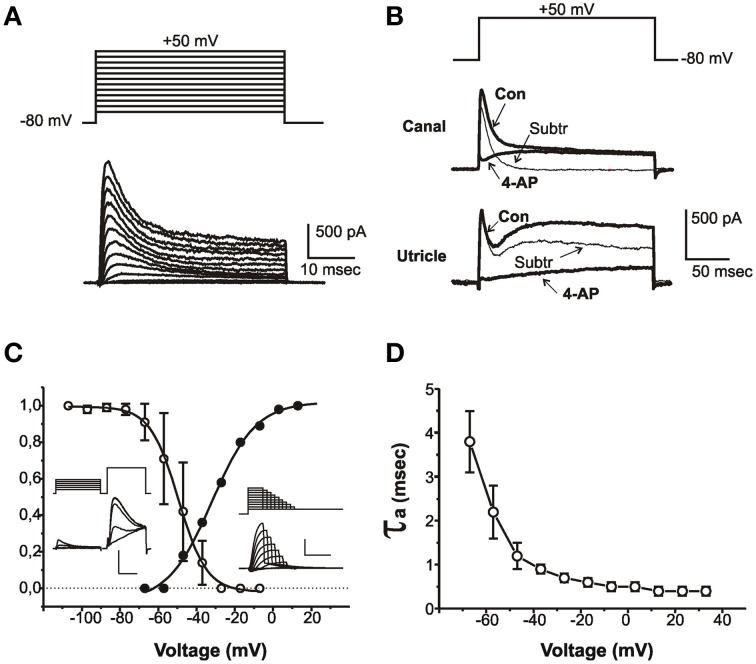
**Properties of I_A_**. **(A)** Current traces evoked from an sB hair cell with 40 ms-depolarizing steps from a holding potential of -80 mV. Voltage protocol is shown above. **(B)** Effect of 4-AP on outward currents from a club-shaped semicircular canal hair cell (top traces) and from a utricular eB hair cell (bottom traces). Thick traces represent control currents (Con) recorded in low-Ca to abolish Ca and Ca-dependent currents, and in low-Ca plus 15 mM 4-AP (4-AP). Thin traces (Subtr) represent subtraction between Con and 4-AP traces. Voltage protocol is shown above. **(C)** Voltage-dependence of I_A_ activation and inactivation. Insets show representative traces of the protocols used for measuring inactivation (left traces) and activation (right traces). The test step in the left inset is expanded to better display I_A_ inactivation. Vertical calibration bars: 400 pA. Horizontal calibration bars: 5 ms for activation and test steps; 50 ms for conditioning steps. **(D)** Voltage-dependence of I_A_ activation time constant (*n* = 8). Current activation was fitted with a second-order HH equation; currents were evoked from a holding potential of −80 mV to partially inactivate I_Na_.

Inactivation curves were obtained by conditioning the cell at various potentials, and then measuring currents with opportune steps. I_A_ activation and inactivation protocols were performed in the presence of RLC to eliminate contributions by BK currents. However, since I_Kva_ could not be blocked pharmacologically without affecting I_A_ as well, a contamination by its activation was usually seen as a second activation component at more positive potentials. To minimize this problem, I_A_ activation was measured in cells displaying small I_Kva_, and I_A_ inactivation values were obtained by subtraction of the trace conditioned at -20 mV (see Figure 1C), when all the transient component was inactivated and a single kinetic component of activation (corresponding to I_Kva_) was left. Data significance was tested with ANOVA or Student's *t*-test. *P* < 0.01 was considered highly significant and *P* < 0.05 significant. In all figures and text, data are represented as mean ± standard error (SE). Error bars in figures represent one SE.

### Capacitance measurements

Whole-cell capacitance (Cm) was monitored by setting the Optopatch amplifier in the track-in mode (Johnson et al., 2002). After a partial manual compensation of series resistance (Rs) and Cm, 50 mV peak-to-peak voltage sinusoids at 1.5 kHz were superimposed on a nominal holding voltage of −90 mV, and Rs and Cm controls were manually adjusted to minimize the sinusoidal component of the whole-cell current. At this point, the built-in lock-in amplifier was turned on, the phase was manually optimized, and capacitance and resistance dithering circuits were activated to calibrate the system. The track-in feedback circuit was then switched on, and its gain was gradually increased to its highest stable value (usually 20–50). Cm was recorded for 60 s at −80 mV to monitor baseline stability. In order to evoke release, hair cells were stimulated with 50–1000 ms depolarizing steps to −60/0 mV, during which the lock-in signals were gated out. Cm traces were recorded at 1 kHz and filtered online at 150 Hz or lower if needed. After depolarizations of variable length and amplitude, Cm variations were calculated as the difference between average Cm values before and after steps. The size of vesicular pools was estimated by assuming a unit vesicle capacitance of 37 aF (Lenzi et al., 1999).

### Model

Biophysical parameters obtained from the present experiments and literature were used to build a NEURON model of eB and F frog utricular hair cells synaptically connected to an afferent. Our hair cell model contained several types of ion conductances (mechanosensitive, passive, voltage- and Ca-dependent), plus a Ca-buffering system, and was connected to a passive afferent through a ribbon synapse. In the afferent, no geometrical effects of arborization were considered, and a single synapse was present.

MET currents were modeled as in Shepherd and Corey (1994). The range of displacements considered was between 0.1 and 2 μm, since displacement-response curves (DRCs) in hair cells from the frog utricle were found to be < 2 μm (Baird, 1994b). To account for hair bundle geometrical variations, correction factors were added to DRC and gmax (Baird, 1994b). Reversal potential of endolymph K was assumed to be 0 mV: to simplify calculations, endolymphatic/hair bundle K was considered to be a different ionic species from basolateral K.

Frog utricle hair cells expressed the following basolateral currents: IA, IKv, IKCa, IKir, INa, Ica, and passive leak. Each current (except for leak, which was linear) was modeled using an equation of the type:

$$\begin{array}{l} I_{s}=\bar{g}.\sum \left(O\right).ghk\left(s_{i},s_{o},v_{m}\right) \end{array}$$

Where *s* is the carried ion, *gbar* the maximal conductance, Σ(O) the sum of open states of the relative kinetic model, *ghk* the Goldman-Hodgkin-Katz current equation for the ion *s, s*_*i*_*, s*_*o*_ the intracellular and extracellular concentrations of *s*, and *v*_*m*_ the membrane potential.

I_A_, I_Kv_, and I_KCa_ consisted of several components: in particular (see patch clamp data), I_A_ displayed a fast and a slow component in type B cells, similar to crista hair cells (Russo et al., 2007); I_Kv_ was the sum of a capsaicin-sensitive component (I_Kvc_) and a 4-AP-sensitive one (I_Kva_); I_KCa_ displayed a rapidly inactivating and a noninactivating fraction, as in other frog hair cells (Masetto et al., 1994; Armstrong and Roberts, 2001). Slow inactivation of BK currents, which was also observed, was assumed to reflect Ca^2+^ dynamics and was not explicitly introduced in the model.

Models for I_Kva_, I_Kvc_ and I_KCa_ were taken from Catacuzzeno et al. (2003), the model for IK1 was adapted from Matsuoka et al. (2003) and models for I_Na_ and I_A_ (fast) were adapted from Nigro et al. (2011) and Zagotta et al. (1994), respectively. Parameters for each kinetic model were obtained by fitting pharmacologically- or kinetically-dissected currents with NEURON fitting routines.

First-harmonic frequency-dependent phase and gain values for real and simulated parameters were obtained by fitting the relative traces with a sine function of similar frequency as the stimulus current.

Since afferent resting discharge is observed at voltages where macroscopic Ca currents are negligible, and given that, in hair cells, the opening of one or few Ca channels is sufficient to support vesicle release (Brandt et al., 2005), in order to obtain resting irregular quantal discharges we modeled voltage-dependent stochastic single-channel Ca currents. To simulate active zone Ca^2+^ entry, and to minimize computational instability in the model when v was free to vary for long times (up to 10 s), deterministic whole-cell Ca currents were used to obtain voltage traces, which were in turn employed to evoke stochastic Ca currents linked to vesicular release. We modeled active zones as having 100 channels [which is the average presynaptic population at a single active zone found in other hair cells (Roberts et al., 1990; Wittig and Parsons, 2008)] and simulated Ca-dependent glutamate release by modifying a stochastic ribbon synapse model (Sikora et al., 2005) in order to linearize Ca-dependence and remove presynaptic fatigue.

Hair cell cytosolic [Ca^2+^] was simulated by assuming accumulation due to inflow through voltage-dependent Ca channels and removal due to a mobile Ca buffer (as in Roberts, 1994). Buffer properties were matched to literature data available for the utricle (Baird et al., 1997); I_Ca_ inactivation (Schnee and Ricci, 2003) was only added to macroscopic currents, and intracellular store release (as in Lelli et al., 2003) was not considered. Postsynaptic response were simulated by modifying the retinal ribbon synapse model by Sikora et al. (2005). In order to isolate the effects of presynaptic dynamics, release, and postsynaptic mechanisms were similar for F and eB models. Model files are available upon request to the Authors.

## Results

### Voltage and Ca-dependent currents in frog utricle hair cells

#### I_A_

I_A_ (Figure 1) was found in most utricular hair cells (91/113). This current was completely blocked by 15 mM 4-AP; however, since several utricular hair cells also expressed a 4-AP-sensitive delayed rectifier (see below), a pharmacological dissection as performed in the frog crista could not be used (Figure 1B), and care was needed to separate this component on a purely kinetical basis (see Materials and Methods).

The pharmacological and biophysical properties of the utricular I_*A*_ were similar to those of the frog crista I_A_ (Masetto et al., 1994; Russo et al., 1995; see also Figure 1B).

**Figure 2:**
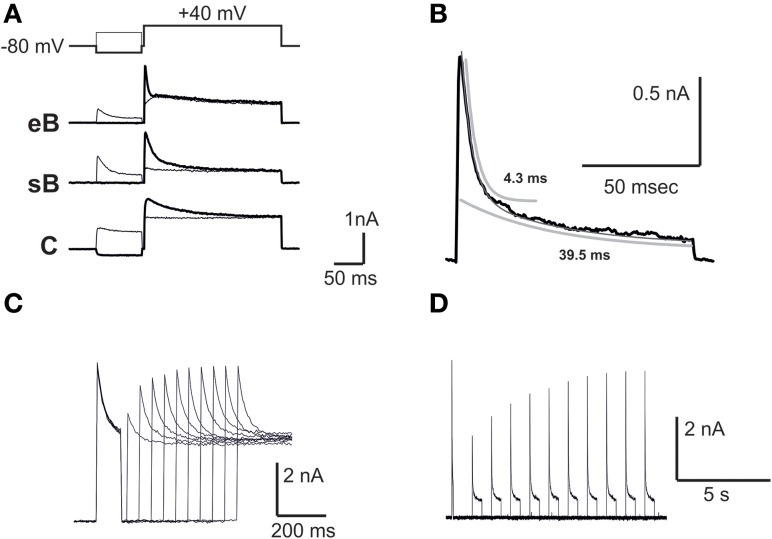
**I_A_ inactivation. (A)** Currents recorded from typical type eB, sB, and C cells in response to a voltage conditioning protocol (shown on top). I_A_ was obtained from subtraction between currents with and without prepulse. Monoexponential fits of I_A_ decay in the traces shown here gave time constants of 5.3 ms (eB), 18.6 ms (sB), and 79.8 ms (C). **(B)** Biexponential fit in a cell expressing both fast and slow I_A_. Time constants are shown on gray fit lines. **(C,D)** time-dependent recovery of slow **(C)** and fast **(D)** I_A_. Protocol consists in a depolarizing step at +20 mV followed by variable time at -80 mV and a second step at the same potential.

I_A_ started activating around −60 mV (Figure 1C, solid symbols) and its V_½_ was −31.8 ± 1.1 mV (*n* = 5); activation time constants were voltage-dependent (Figure 1D). I_A_ inactivation was completely removed at −100 mV and half-completed at −44.1 ± 0.4 mV (*n* = 8) (Figure 1C); I_*A*_ displayed noticeable window currents between −60 and −30 mV. I_A_ inactivation timecourse (Figure 2) followed two time constants: τ_f_ = 5.6 ± 0.5 ms (*n* = 8) and τ_s_ = 36.4 ± 4.6 ms (*n* = 4) (Figure 2B). Different cells displayed different proportions of rapid and slow I_*A*_ (Figure 2A). The rapidly inactivating component recovered slowly (τ = 2.7 s at −80 mV, *n* = 3; Figure 2C), whereas the slowly inactivating one recovered much faster (τ = 85.7 ± 4.5 ms at −80 mV, *n* = 3; Figure 2D). Utricular I_A_ currents displayed similar features as the I_A_ from the frog crista (Norris et al., 1992; Russo et al., 2001, 2007). During step protocols, interpulse times needed to be longer than 30 s, since shorter intervals induced a partial inactivation of the fast I_A_: therefore, this component displayed cumulative inactivation for stimulation frequencies down to 0.03 Hz.

#### I_BK_

**Figure 3:**
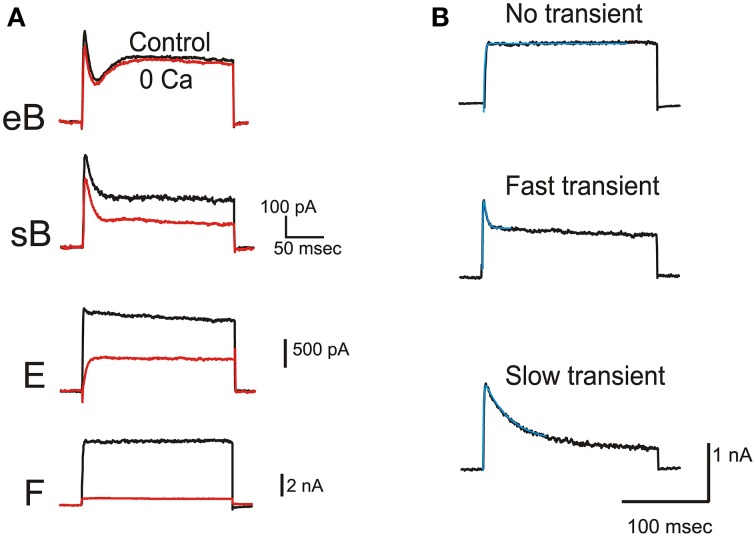
**BK current. (A)** Effects of extracellular Ca removal on typical eB, sB, E, and F hair cells. Current traces were evoked by 200 ms-depolarizations to 0 mV. **(B)** Inactivation kinetics of Ca-dependent currents. Traces represent subtractions between control and 0 Ca traces obtained by protocols as in **(A)**. Activation could be fitted with a single exponential in all cells (here τ_a_ were 0.99, 0.82, and 0.79 ms from top to bottom). Inactivation was absent from type F cells (top panel). In other cells, fast (middle panel, τ_i_ = 2.97 ms) and slow (bottom panel, τ_i_ = 30.23 ms) components of inactivation were seen. Fits are indicated by thin blue traces.

Ca-dependent currents in frog utricle hair cells (Figure 3) were similar to BK currents described in hair cells from the frog canal (Masetto et al., 1994) and saccule (Armstrong and Roberts, 2001). I_BK_ activated rapidly (τ_a_ = 0.96± 0.23 ms at 0 mV, *n* = 9) and represented the major steady outward current in striolar hair cell types, especially in non-B cells (Figure 3A). In type F cells (Figure 3B), I_BK_ did not inactivate, similar to that observed in pear-shaped cells from the semicircular canal (Prigioni et al., 1996), or inactivated very slowly. In other cell types, I_BK_ displayed transient and steady components, as seen in canal and saccule hair cells (Masetto et al., 1994; Armstrong and Roberts, 2001). In addition to the rapid inactivation described in saccular hair cells, a slower component of inactivation could also be observed in some cases (Figure 3B). Both fast and slow components could be present within the same cell (not shown).

#### I_Kv_

**Figure 4:**
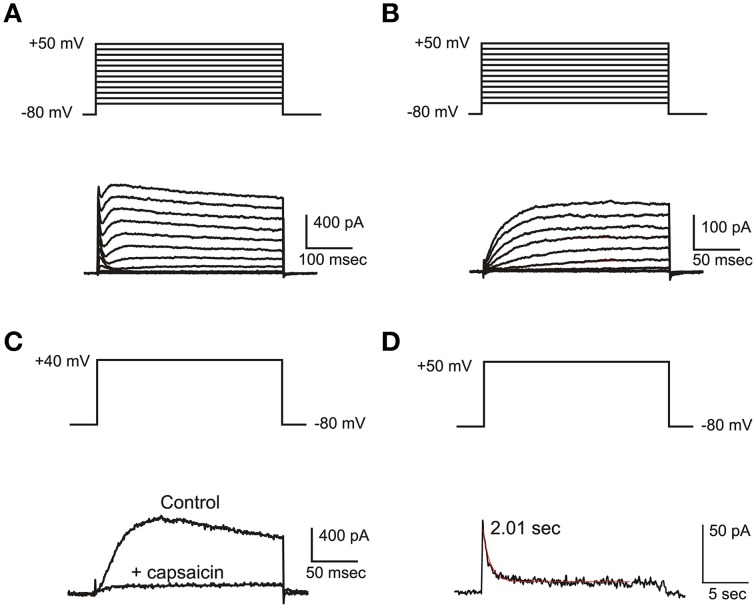
**IKv**. **(A)** Current families evoked from an eB hair cell. A fast I_A_ and a slower delayed rectifier are evident. **(B)** Activation of slow delayed rectifier current (I_Kvc_) from an eB cell. Current traces were evoked in the presence of 15 mM 4-AP and 10 mM TEA. **(C)** Effect of capsaicin. Control: 15 mM 4-AP and 10 mM TEA; capsaicin: same as control plus capsaicin 50 μM. **(D)** slow inactivation of I_Kvc_. Voltage protocols are shown on top of each panel.

I_Kv_ (Figure 4A) in frog utricle hair cells activated at rather depolarized potentials, and could be separated in a 4-AP sensitive component (I_Kva_) and a capsaicin sensitive *one* (I_Kvc_). I_Kva_ started activating around −30 mV, and activated and inactivated slowly (τ_a_ = 12.3 ± 3.4 ms at +43 mV; τ_i_ = 340 ± 72 ms at +43 mV; *n* = 5). After blocking I_A_, I_BK_, and I_Kva_, a residual outward current was observed in most hair cells (Figure 4B). This current activated very slowly at depolarized potentials (τ_a_ = 116.5 ± 12.4 ms at 0 mV; V_1∕2_ = 0.6± 4.1 mV; *n* = 5), decayed in a voltage-independent way following a double exponential (τ_f_ = 370 ± 130 ms; τ_s_ = 3.35 ± 1.28 s; *n* = 4) (Figure 4C), and was blocked by capsaicin 50 μM (Figure 4D), similarly to the slow delayed rectifiers found in the frog crista and saccule; to maintain consistency with the canal nomenclature, it was therefore called I_Kvc_.

#### I_K1_

**Figure 5:**
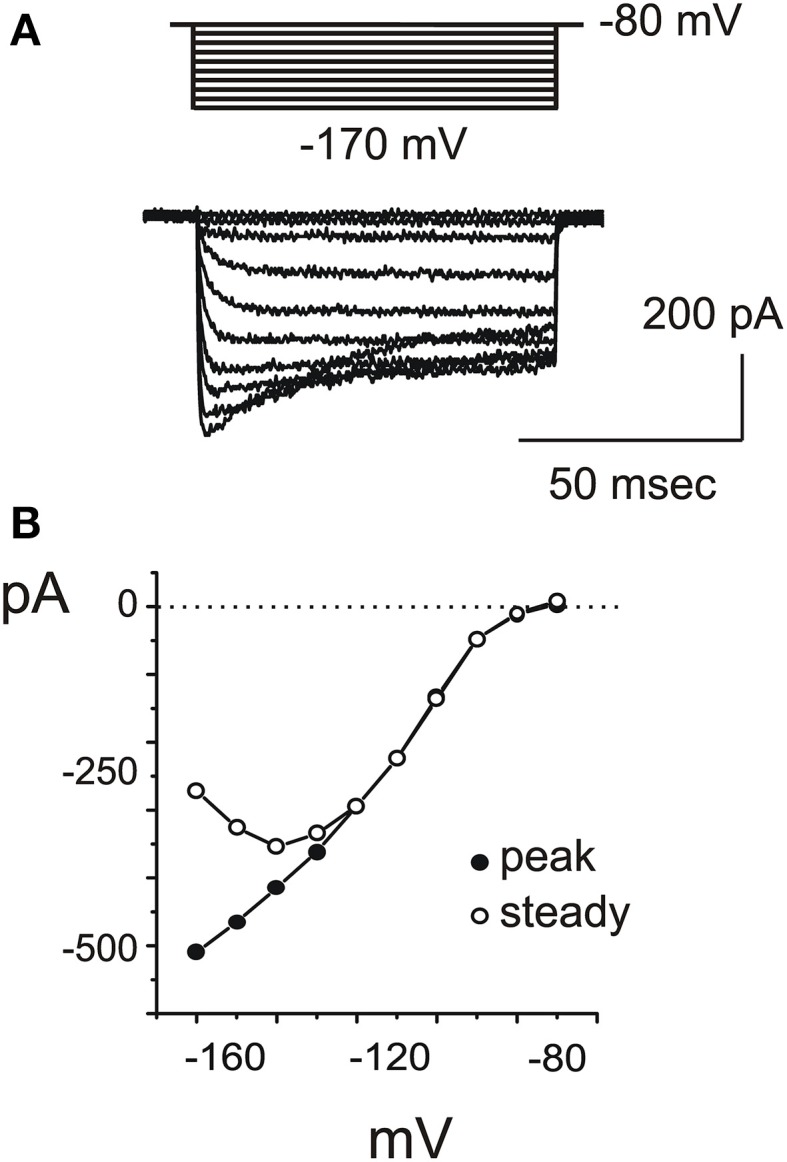
**I_KI_. (A)** Inward rectifier currents evoked in a type F cell with 200 ms steps from −80 to −170 mV. **(B)** Voltage-dependence of IK1. At very negative potentials, partial inactivation was observed (empty circles).

A fast inward rectifier similar to I_K1_ (Figure 5) started to be detectable around −80 mV, activated with a rapid, monoexponential kinetics (τ_a_ = 2.5± 0.7 ms at −100 mV; *n* = 3) and partially inactivated for very negative voltages (Figure 5B). Inward rectifier currents were blocked by 5 mM extracellular Cs. Utricular hair cells never displayed a slow, Ba-insensitive inward component indicative of I_*h*_ (*n* = 107), similarly to the frog crista (Masetto et al., 1994; Russo et al., 1995) but differently from the frog saccule (Catacuzzeno et al., 2003).

#### I_Na_

**Figure 6:**
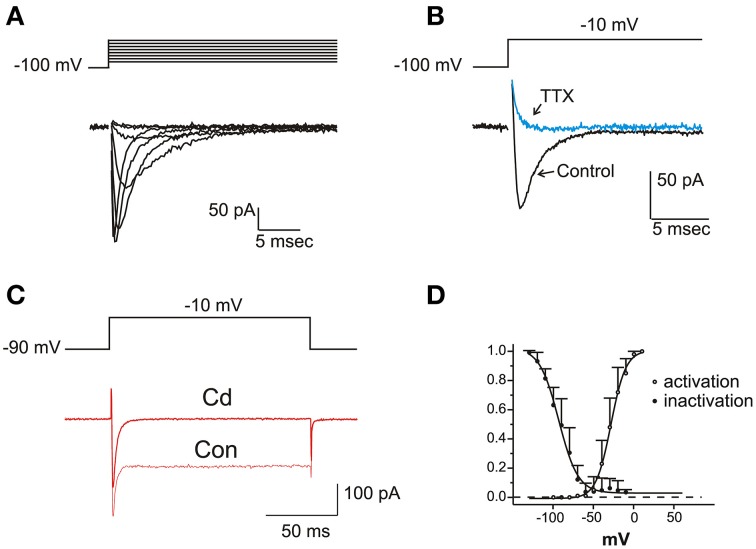
**Voltage dependent Na currents**. **(A)** Current traces recorded from an eB hair cell in the presence of intracellular Cs and extracellular K- blocking cocktail (Ba extracellular, see Materials and Methods) plus Cd 100 μM. Protocol is shown on top. **(B)** Effect of TTX 100 nM. Same conditions as in **(A)**. **(C)** Effect of Cd 200 μM. I_Na_ is not affected, whereas the nondecaying I_Ba_ is completely blocked. Current traces recorded in intracellular Cs and extracellular Ba solutions. **(D)** Voltage-dependence of activation and inactivation. Activation data are derived by tail current measurements at −70 mV following short steps to the indicated potentials; inactivation data are obtained from normalized peak current evoked at −20 mV after a 500 ms- conditioning at the indicated potentials. Activation and inactivation half-potentials from Boltzmann best-fits (indicated as smooth curves) were −28.6 ± 0.7 mV (*n* = 5) and −93.3 ± 1.5 mV (*n* = 9).

In a fraction of utricle cells (57/113), upon depolarization from negative holding potentials (−100 mV), fast-inactivating inward currents were observed after blockade of K^+^ channels with TEA 6 mM, 4-AP 15 mM and capsaicin 50 μM (Figure 6A). These were pharmacologically identified as neuronal voltage-dependent Na^+^ currents, since they were reversibly blocked by nanomolar TTX, and unaffected by 200 μM Cd (Figure 6B) or extracellular Ca removal (not shown). Na^+^ currents were never observed when employing similar protocols and conditions on frog saccular (*n* = 42) or canal (*n* = 77) hair cells.

Na^+^ currents activated for potentials more positive than −60 mV and peaked between −30 and −20 mV (Figure 6C). Steady state inactivation was complete at −50 mV, and completely removed at −120 mV (V_½_: −81.6 ± 1.4 mV; *n* = 6); no significant window current was found at any potential. Activation and inactivation time constants were strongly voltage-dependent, both decreasing about 10-fold between −30 and +30 mV (τ_a_: from 1.08 ± 0.38 to 0.11 ± 0.05 ms; τ_i_: from 4.03 ± 0.93 to 0.41 ± 0.15 ms; *n* = 6).

#### I_Ca_

**Figure 7:**
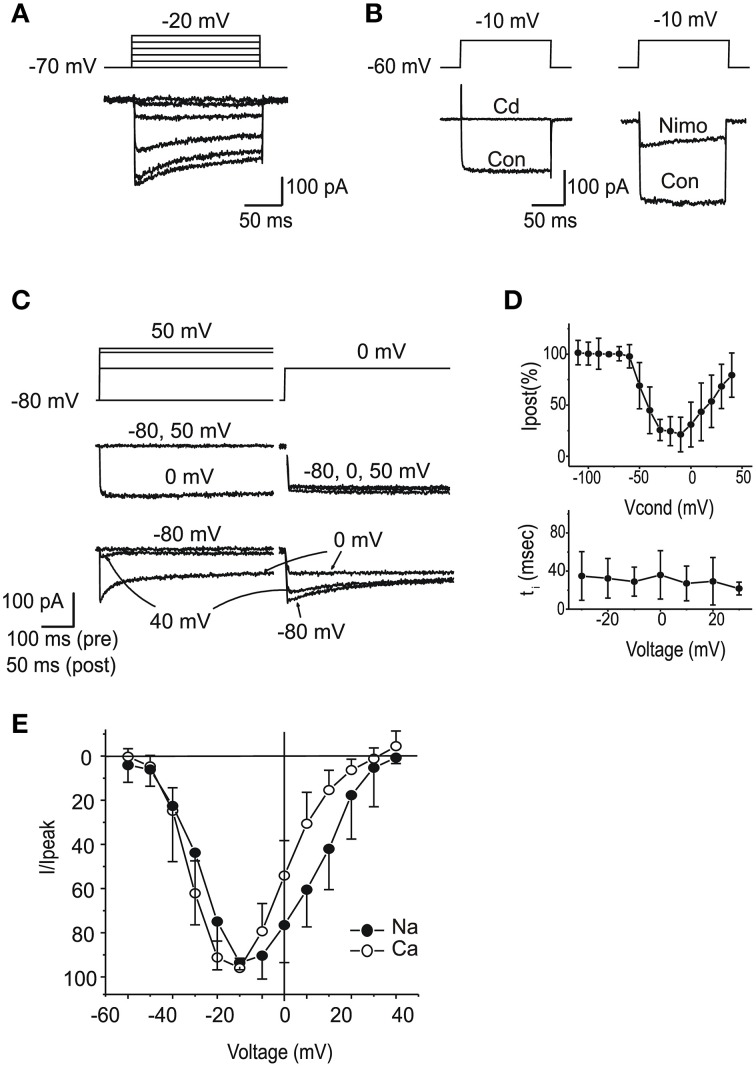
**Voltage dependent Ca currents**. **(A)** Currents evoked in intra-Cs, extra-Ba solutions with 100 nM TTX. Note the presence of voltage-dependent inactivation. **(B)** Effects of Cd 200 μM (left) and nimodipine 10 μM (right). Same solutions as **(A)**. **(C)** Ca-dependent inactivation. 500 ms conditioning steps at variable potentials were followed by a test step. In the presence of 5 mM Ca (red traces), inactivation of the test step was maximal for potentials that evoked the largest current in the conditioning step. In the presence of Ba 5 mM (black traces) Ca-dependent inactivation was abolished. **(D)** Voltage-dependence of Ca-dependent inactivation fraction (*n* = 5, upper panel) and timecourse (lower panel) **(E)** I-V plot for normalized inward currents in eB type hair cells (*n* = 10). Filled circles, Na currents; Open circles, steady-state Ca currents from the same cells. Ca currents are obtained with protocols as in **(A)**, from a holding potential of –60 mV; Na currents are obtained in the presence of Cd or as subtraction between currents from holding potentials of −100 and −60 mV.

After blocking K^+^ currents, the majority of utricular hair cells displayed fast inward currents upon depolarization. From a holding potential of -60 mV (Figure 7A), currents observed in the presence of 5 mM Ca and Ba were similar to those described in the frog saccule (Armstrong and Roberts, 1998; Rodriguez-Contreras and Yamoah, 2001) and semicircular canal (Martini et al., 2000; Perin et al., 2001; Russo et al., 2001). Currents were increased by the substitution of Ba for Ca, strongly reduced by 10 μM nimodipine (Figure 7B), and completely blocked by Cd 200 μM (Figure 7B). In about half the cells, I_Ca_ displayed inactivation, comprising a Ca-dependent component (Figures 7C,D), and a second component that persisted in Ba (see Figure 7A). Ca currents activated around −60 mV, and peaked at −20 mV, similarly to Na currents (Figure 7E).

#### Hair cell classification and current expression

**Figure 8:**
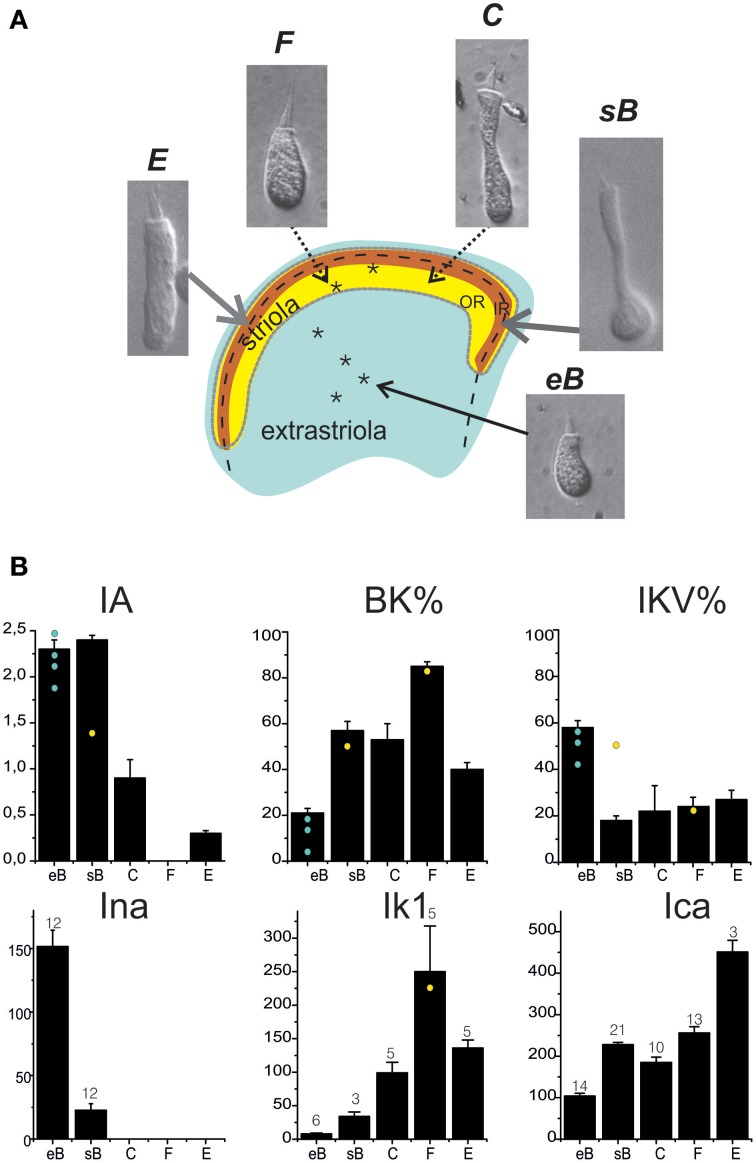
**Hair cell types in the frog utricle**. **(A)** Schematization of the frog utricular macula showing the striolar region (outer striola (OR): yellow, inner striola (IR): orange) and cell types located in each region. Inset panels: Nomarski interferential microphotographs of isolated hair cells from the frog utricle. Cells are labeled following the hair bundle-based classification used in Baird (1994b). Extrastriolar cells are small, short and with a narrow cuticular plate, few short stereocilia, and a long kinocilium (type eB). In the striola, four different cell types (sB, C, E, F) are found. Striolar type B cells display a similar hair bundle as extrastriolar cells but their round body terminates in a long narrow neck. Types C (elongated) and F (rounded) cells have larger hair bundles and cell bodies (between these two types, several intermediate types were found). Type E cells are clearly distinguishable by their large cylindrical body and bulbed kinocilium, similar to saccular hair cells. Asterisks mark the approximate location of hair cells recorded from *in situ* preparations. Extrastriolar cells all gave an eB response (not shown); the two striolar responses were C (not shown) and F-type (black traces). Voltage steps were at −120, −60, 0 mV from a holding potential of −60 mV. Scale bars: 50 ms, 200 pA. Note the absence of inactivating currents. **(B)** Distribution of basolateral currents in different cell types. BK and IKV are given as percentage of steady currents at 0 mV, all other currents as actual amplitudes measured at selected voltages (ICa, INa: −20 mV; IA: +20 mV. IK1: −120 mV). Cell numbers are indicated for each histogram. Yellow spots and blue spots indicate currents recorded from *in situ* hair cells.

Utricular hair cells (Figure 8A) were morphologically classified following their hair bundle and soma shape (Baird, 1994b). The morphological classification appeared to hold for current expression as well (Figure 8B). Type B cells displayed a predominant fast I_A_, a large delayed rectifier, a small I_Ca_ and I_BK_, and I_Na_. Extrastriolar type B cells displayed larger I_Na_ and I_Kva_ and smaller I_BK_ than striolar cells. Type F cells displayed a major I_Ca_-I_KCa_ system and often a fast I_K1_ inward rectifier. Type C cells displayed the same currents as type F plus a slow I_A_ and a small delayed rectifier. Type E cells, which are coupled to vibratory afferents, expressed similar currents as saccular hair cells, with the exception of I_h_. Most current data were obtained from trypsin-isolated hair cells, to ensure good pharmacological access and cell shape recognition; recordings from hair-bundle identified *in situ* hair cells (*n* = 6, asterisks in Figure 8A) gave currents not significantly different from those recorded from isolated hair cells. *In situ* recordings were performed as control, to ensure that trypsin digestion did not alter current expression, and were not enough to build a gradient map of current expression.

#### Voltage responses

To study the roles of basolateral ion channels in hair cell stimulus processing, we recorded voltage responses to step and sinusoidal current stimuli in extrastriolar B type and striolar F type.

#### eB-type cells

**Figure 9:**
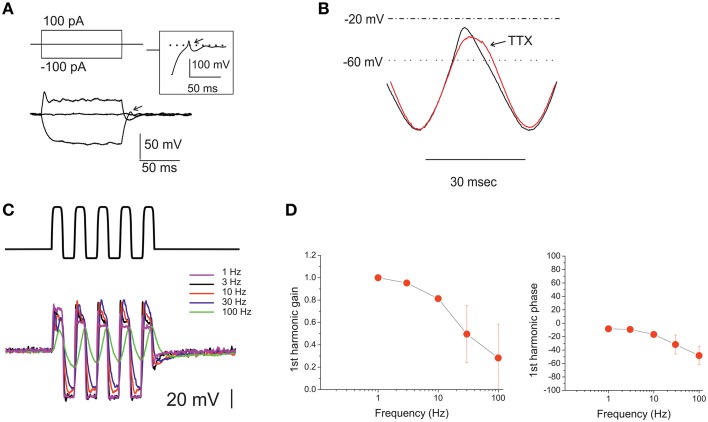
**Voltage responses of utricle eB hair cells**. **(A)** voltage responses of an eB cell to the currents indicated in the protocol above. Dashed line indicates 0 mV. Inset: repolarization phase from a different eB cell, showing a spikelet. Arrows indicate active responses during return from hyperpolarization. **(B)** Voltage response of an eB cell to a ±100 pA current sinusoid at 33 Hz. Black trace, control; red trace, TTX 100 nM. **(C)** voltage responses of an eB cell to ±100 pA current sinusoids at different frequencies (1–100 Hz) indicated in color to the right of the traces). **(D)** Bode plots for first-harmonic frequency-dependent responses (*n* = 5).

Resting potential for eB cells was −61.6 ± 2.1 mV (*n* = 16). Depolarizing current steps elicited a small peak followed by a plateau, whereas hyperpolarizing current steps were passive and followed by active overshooting responses (Figure 9A).

The morphological properties of type B hair bundles (long kinocilium, few short stereocilia) suggest that MET currents in these cells are small (Baird, 1994a). However, due to the absence of inward rectifiers and to the depolarized activation of their delayed rectifiers, eB cells display a very large input resistance, and therefore even small MET currents can evoke receptor potentials large enough to activate Ca currents (which are of similar nature as in other utricular hair cells). Without any corrective mechanism, however, this input resistance would dramatically slow down the cell voltage response: even in these small cells (Cm: 3.8 ± 0.2 pF; *n* = 37), the measured input resistance (4.2 ± 0.6 GOhm) would filter voltage signals with a time constant of 16.1 ± 1.1 ms. Depolarizing responses, however, are much faster, and upon return from hyperpolarization, the kinetics went from exponential to active, in some cases with a full rebound spikelet (Figure 9A, inset). Part of the active response is due to I_Na_ recruitment, since TTX reduces and slows depolarization after hyperpolarization (Figure 9B). It is interesting to note that I_Na_ activation voltage and timecourse are similar to those of I_Ca_ (see Figure 7D) and therefore this current helps depolarizing hair cells within the voltage range important for neurotransmitter release, without loading the cells with Ca (process that would be especially dangerous in the small, buffer-poor extrastriolar type B hair cells; Baird et al., 1997).

Upon sinusoidal stimulation between 0.3 and 100 Hz (Figures 9C,D), first-harmonic voltage responses showed a clear gain roll-off (−3 dB corner at 52.5 ± 12.8 Hz, *n* = 5), and a moderate phase lag (48.1 ± 13.5 at 100 Hz; *n* = 5) at higher frequencies.

#### F-type cells

**Figure 10:**
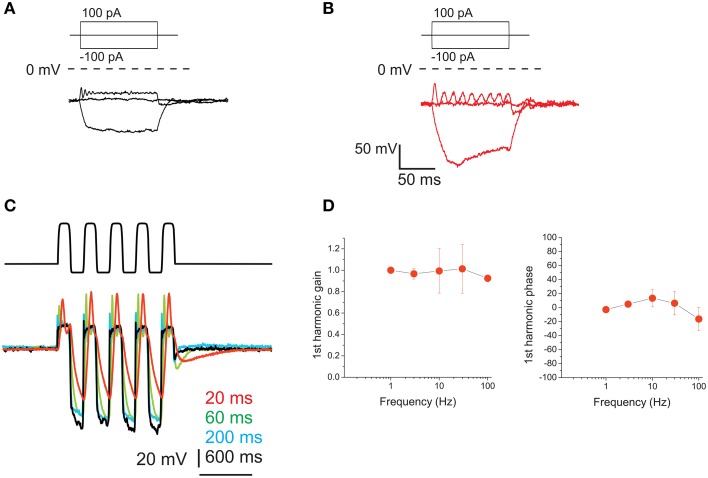
**Voltage responses of utricle F hair cells**. **(A)** voltage responses of an F cell to the currents indicated in the protocol above. Dashed line indicates 0 mV. For this cell, resonant frequency was 118 Hz and Q factor was 4.9. **(B)** Resonant response of a saccular hair cell. Same conditions and protocol as **(A)**. Resonant frequency: 48 Hz. **(C)** Voltage responses of an F cell to ±100 pA current sinusoids at different frequencies (indicated in color to the right of the traces). **(D)** Bode plots for first-harmonic frequency-dependent responses (*n* = 4).

Resting potential for type F cells was −64.5 ± 1.3 mV (*n* = 6). Type F cells displayed high-quality electrical resonance upon step depolarization (Figures 10A,B), at frequencies higher than those predominant in head motion. This resonance induced a frequency-dependent gain increase in voltage responses. Resonant frequencies and Q of type F cells were 60 ± 46 Hz and 4.7 ± 0.3 (*n* = 4); to test whether its properties were altered by our dissociation protocol, similarly to what was found in the frog saccule for papain (Armstrong and Roberts, 1998) we recorded from saccular hair cells as well (Figure 10B) which displayed responses similar to *in situ* hair cells, as previously observed with trypsin (Catacuzzeno et al., 2003). Hyperpolarizing current steps elicited small responses consistent with the expression of fast inward rectifiers (Figure 10A) but never induced sags consistent with Ih, which were instead found in saccular hair cells (Figure 10B).

Upon sinusoidal stimulation, depolarization amplitudes increased with frequency (Figure 10C) (although the increase was limited for the 1st harmonic component, see gain in Figure 10D), as opposed to eB cells. However, phase responses were small: phase leads were observed at 10 and 30 Hz, but at 100 Hz they were not always present, and small phase lags could be observed instead.

#### Release

**Figure 11:**
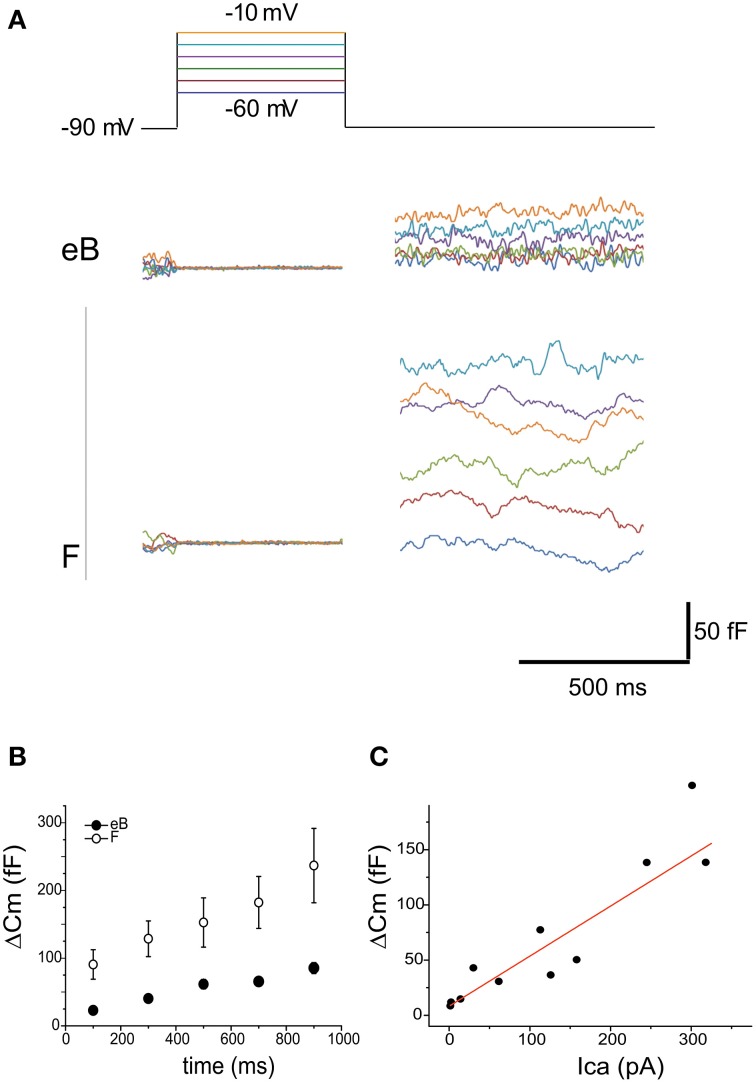
**Release**. **(A)** capacitance increases evoked in eB and F cells with 500 mV voltage steps to various potentials (color coded in the top panel). Cm increases display the same voltage dependence as Ca currents. Note the larger size of Cm increase in F cells vs. eB cells. **(B)** Cm steps were evoked with depolarization at −10 mV from 100 to 900 ms. No clear adaptation was observed in time either in eB (blue, *n* = 6) or in F cells (red, *n* = 4); **(C)** Ca-dependence of Cm increases is linear (*R*^2^ = 0.89). Data are taken from both B and F hair cells (*n* = 6) at different times and voltages of stimulation.

In order to observe whether release nonlinearities could affect utricular hair cell output, we measured ΔCm in frog utricle hair cells (Figure 11). Releasable pool size was smaller in B cells (139.5 ± 129.4 fF, 100 ms at −20 mV, *n* = 6; Figure 11A) than in F cells (361.4 ± 145.1 fF, 100 ms at −20 mV, *n* = 6; Figure 11B) paralleling differences in Ca current amplitudes (104 ± 5 pA in eB cells, *n* = 15; 256 ± 15 in F cells, *n* = 15). In both cell types, release did not display adaptation for stimuli as long as 1 s (Figure 11B) and followed Ca entry linearly (Figure 11C), as observed at other ribbon synapses. To ensure that recording conditions did not change during ΔCm measurements, Ca currents were recorded at the beginning and at the end of experiments. Cells where Ca current amplitude changed more than 20% during the experiment were rejected since in perforated patch condition Ca currents are known to be stable (Martini et al., 2004), and therefore a change in amplitude could be associated to spontaneous patch rupture or other factors impairing intracellular Ca homeostasis.

#### Model

**Figure 12:**
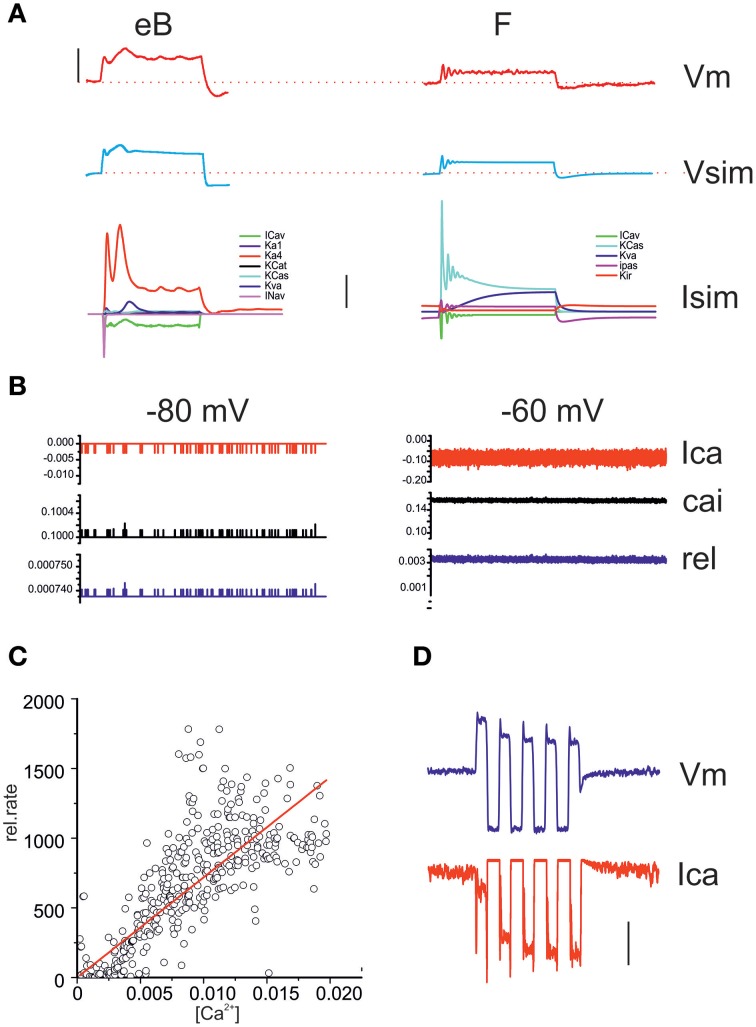
**Model responses. (A)** Comparison between real voltage responses to 100 ms, 100 pA step currents and simulation with eB (left) and F cell (right) models. Red, recordings; blue, simulations. Vertical scale bar: 40 mV. Stippled lines indicate −60 mV. The lowest panel displays the model currents activated during the depolarizations shown in the top traces. Vertical scale bar: 200 pA. **(B)** Simulated Ca currents (red) intracellular Ca increases in release nanodomains (black) and instantaneous release probability (blue) from a resting potential of -80 and -60 mV (1 s). **(C)** Correlation between instantaneous Ca concentration (x axis) and instantaneous release rate (y axis) in a 1 s long simulated release trace. A linear regression was fit to the data (*R*^2^ = 0.88). **(D)** Simulation of an eB response to a 100 pA, 1 Hz sinusoidal current. The second depolarizing half-cycle (measured *re*. resting average) was 67% of the first for the voltage response (blue) but 178% for the calcium increase (red). Vertical bar: 50 mV (Vm); 50 pA (Ica); 5 kHz (release).

A NEURON model was built of eB and F hair cells, containing all currents found for each cell type, with activation and inactivation properties fit to recorded voltage-clamp data. Model cells (Figure 12) reproduced hair cell voltage responses to current clamp protocols. In type eB model, resting potential is dominated by the IA window current (which balances 86% of depolarizing passive leak at −62 mV) whereas in type F model resting potential is dominated by IK1 (which is the only voltage-dependent player below −60 mV). All other currents are activated upon depolarization. The contribution of each current to voltage responses is shown in the lowest panel in Figure 12A. In order to estimate the contribution of voltage modulation to postsynaptic dynamics, we simulated release in eB and F models. Release was driven by the opening of single stochastic voltage-dependent Ca channels with the same voltage dependence as whole cell I_Ca_. In both hair cell models, the synapse contained 100 Ca^2+^ channels and 45 release sites. This model gave an average instantaneous release probability of 0.0007 ± 2e-7 and 0.003 ± 9e-5 from hair cell potentials of −80 and −60 (Figure 12B), which encompass all measured resting potentials. Model release increased linearly with intracellular Ca (Figure 12C) similarly to what was observed experimentally (see Figure 11). On the other hand, voltage responses to large currents (100 pA or more) crossed ICa activation peak voltage, and the progressive decrease of depolarizing responses seen for low-frequency stimuli could actually generate a progressive calcium entry increase (Figure 12D).

**Figure 13:**
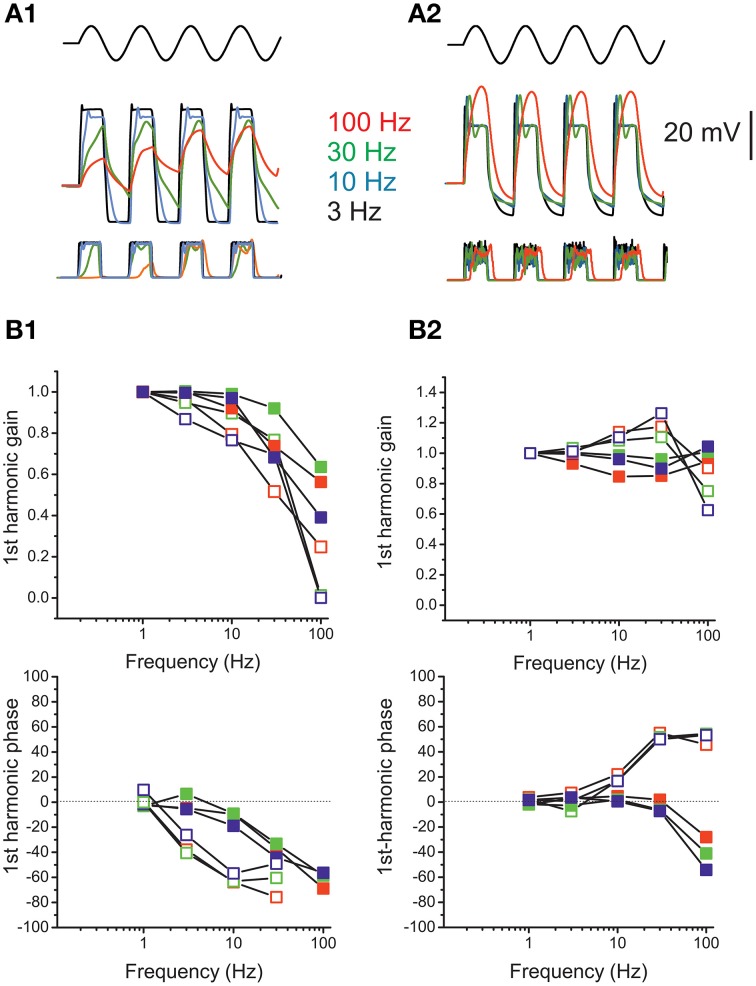
**Response dynamics of simulated release**. **(A)** Simulated response of an eB cell **(A1)** and an F cell **(A2)** to sinusoidal mechanical stimulation (3–100 Hz, ±2 μm). Top traces, hair bundle movement; Middle traces, voltage responses; bottom traces, instantaneous release rates. Colors indicate frequencies. **(B)** Bode plots showing, for eB **(B1)** and F **(B2)** cell model, the frequency dependence of first-harmonic phase and gain of voltage (red symbols) Ca current (green symbols) and release rate (blue symbols). Solid symbols, responses to 2 μm hair bundle oscillations; empty symbols, responses to 0.5 μm hair bundle oscillations.

The model was used to quantify frequency-dependent gain and phase behavior of release in eB and F utricular hair cells upon mechanical stimulation. For the eB cell model (Figures 13A1,B1), membrane voltage displayed low-pass behavior, more evident for small stimuli. Calcium and release displayed the same frequency-dependence as voltage for small stimuli, but were much less reduced at high frequencies for saturating stimuli, given that depolarizations crossed I_Ca_ peak voltage. As regards phases, Ica, and release mechanisms did not introduce significant shifts.

In the F cell model (Figures 13A2,B2), depolarization displayed a frequency dependent gain increase and phase lead for small stimuli, but this pattern was not maintained for saturating stimuli, where frequency-dependent lags and frequency-independent gain were instead observed.

Model responses show that realistic voltage dynamics, although displaying large phase and gain difference between phasic and tonic cells, is not sufficient to impart the observed dynamic features into the postsynaptic response, when coupled to ribbon synapse release sustained by single L-type channels.

## Discussion

In the present work we characterized ionic currents, voltage responses and release properties in hair cells from the frog utricle, and built a realistic NEURON model of these cells. Our results show that, in the frog utricle, the voltage responses of striolar type F cells have a different dynamics from extrastriolar B cells, paralleling the dynamics of afferents contacting them. In both cell types, release was found to be linearly related to Ca currents. The model allowed us to compare hair cell responses at the level of voltage, presynaptic Ca and release, and showed that voltage dynamic features, although correlated to postsynaptic dynamics, are not sufficient to explain it. In particular, although phase differences between eB and F cells (both in recordings and model) exceeded 90° at frequencies above 30 Hz, the difference was not due to a frequency-independent encoding in tonic cells and a strong phase lead in phasic cells; instead, both cell types displayed frequency-dependent phase shifts in their voltage response. This suggests the need of an additional frequency-dependent, phase-advancing step common to all utricle hair cells in cascade with voltage modulation.

In the remainder of the discussion we will discuss possible roles of basolateral current and synaptic release properties in shaping voltage dynamics in correlation with available data on afferent physiology.

In particular, we will compare our results with those obtained in the frog crista, in order to compare mechanisms in otolithic vs. canal organs, and in the turtle crista, since our voltage- and current clamp experiments were similar to those performed by Brichta et al. (2002) but our results and conclusions are significantly different.

In the frog utricle, extrastriolar type B cells contact tonic afferents, whereas striolar type F cells contact phasic fibers (Baird and Lewis, 1986). This parallels peripheral and pear cells in the frog crista (Honrubia et al., 1989), and planum and torus type II hair cells (or planum type II and type I hair cells) in the turtle crista (Eatock et al., 2006), respectively, since in both cases, tonic fibers display regular, low-pass responses, and phasic fibers an irregular, phase-advanced response. In a previous study of turtle crista hair cells (Goldberg and Brichta, 2002), the contribution of ionic currents to response dynamics was found to be negligible: in all hair cells, voltage displayed poor (*Q* < 2) or no resonance, and small (< 30°) or no phase leads, and contributed in a limited way to the frequency-dependent phase leads measured in afferents (up to 90°). Our study finds similar phase shifts in phasic cells, but a stronger resonance, and an additional frequency-dependent behavior in tonic cells, in the frog utricle. The reasons for this difference may be several.

One possibility is that processing differs between frog and turtle, due to the appearance of type I hair cells in the latter. However, this appears unlikely, since in the rat saccule, type I electrical properties are strongly correlated to postsynaptic dynamics (Songer and Eatock, 2013) whereas in the toadfish crista, hair cell currents do not contribute significantly to it (Rabbitt et al., 2005). In the rat saccule, moreover, immature type I hair cells display electrical tuning, sustained by a Ca-dependent mechanism similar to that found here in type F cells (and in lower vertebrate auditory hair cells). This could therefore represent a primitive mechanism for high-frequency gain increase and phase lead; it appears interesting that both F cells in the frog utricle (Baird, 1994a) and pear-shaped cells in the frog crista (Honrubia et al., 1989) are connected to highly irregular phasic fibers and located in epitelial regions where type I hair cells appear in reptiles, and could therefore represent their precursors.

On the other hand, there could be a processing difference between semicircular canal and utricle hair cells. To address this issue, we compare currents expressed in the frog utricle (from the present work) and crista (from the literature). The distribution of ionic currents in frog canal and utricle hair cells display several similarities, allowing the definition of *three* cell populations. Peripheral hair cells (canal club cells, utricle eB cells) display a large I_A_, a small, partially inactivating I_KCa_, and a small I_Ca_. Intermediate hair cells (canal pear cells, utricle type F cells) mainly display large noninactivating I_KCa_ and I_Ca_ (and in the utricle an inward rectifier). Central hair cells (canal central cells, utricle type C cells) display large I_Ca_ and I_KCa_, plus a delayed rectifier and an I_A_.

When comparing currents expressed by frog utricle and crista hair cells, several major differences are also noted: (1) delayed rectifiers display different pharmacology and kinetics; (2) hair cells in the periphery express large delayed rectifier currents and I_Na_ in the utricle but not in the crista; (3) hair cells in the crista periphery express inward rectifiers, which are small or absent in utricle peripheral hair cells; (4) utricular type F cells express inward rectifiers, whereas canal pear cells lack it (Prigioni et al., 1996).

Since peripheral responses are tonic, i.e., follow the mechanical stimulus, utricle and canal hair cells would be expected to respond to different stimuli at very low frequencies: otolithic membrane displacement can be maintained indefinitely, whereas cupular displacement decays in tens of seconds, due to endolymph mechanics (Highstein et al., 2004).

Utricular and canal delayed rectifiers activate around −40 and −60 mV, respectively (Marcotti et al., 1999). Therefore, in eB hair cells, small depolarizations from Vz (which is around −60 mV) can persist indefinitely, since the only significant repolarizing current is a largely inactivated I_A_. For larger stimuli, hair cell currents would attenuate responses during the first 500–1000 ms, but after that time all current would largely inactivate.

In the crista, delayed rectifiers are instead activated at rest, and one component (I_Kvb_) does not inactivate even for very long depolarizations (Marcotti et al., 1999), thus providing, together with inward rectifiers, a repolarizing force for small, low-frequency stimuli. Largely noninactivating currents were found in the turtle crista as well, where at least 21% of total current was still present after 60 s depolarization to −47 mV (Goldberg and Brichta, 2002).

Since the inactivation of both delayed rectifiers and Ca currents has been found to be modulated by phosphorylation (Martini et al., 2013), very low-frequency gain could be affected by the efferent system.

The presence of different delayed rectifiers and the selective expression of INa in the utricle and IK1 in the canal could give to tonic cells in the two organs a different phase behavior. INa helps depolarizing utricular eB cells, counteracting the slowing effect of high resting impedence (due to the absence of inward rectifiers and to the depolarized voltage range of delayed rectifiers) and anticipating voltage depolarizing peak. However, at high frequencies, INa would undergo inactivation, thus contributing to the phase lag. In peripheral crista hair cells, resting impedence is lower thanks to IK1 and IKVb expression, which do not display fast inactivation components.

Differences in dynamic behavior are also evident in cells connected to phasic afferents. No voltage response data are available for the frog crista, so comparisons will be made with crista hair cells from different vertebrates. In type F cells, a BK-ICa mechanism for electrical resonance is responsible for their frequency-dependent behavior, similarly to immature rat type I cells (Songer and Eatock, 2013), and nonmammalian auditory cells (including the frog saccule). BK currents are found in most vestibular hair cells, but were notably absent in turtle crista recordings, possibly due to washout of Ca^2+^ buffers during long recordings in ruptured whole-cell patch clamp, or to Ca^2+^ loading due to tonic depolarizations. In fact, depolarizing bias currents were added to turtle crista hair cells with the rationale of ensuring adequate Ca^2+^ entry for resting discharge (Goldberg and Brichta, 2002). However, in the frog amphibian papilla, single vesicles can be released from hair cells at potentials as negative as −80 mV (Li et al., 2009), suggesting that resting potentials measured from isolated cells are compatible with a resting discharge. Accordingly, in our model stochastic single channel openings gave rise to a noticeable resting discharge around −60 mV.

Similarly to type I hair cells, frog utricle F cells are connected to low-gain, phasic afferents (Baird and Lewis, 1986). Our experiments and model could reproduce voltage dynamics with phase leads and gain increase for higher frequencies, but these are smaller than those observed at the postsynaptic side. It is interesting to note, however, that when comparing phasic and tonic release output the difference between the two cell types agree both qualitatively and quantitatively with tose between tonic and phasic afferents.

Further analysis will investigate the effects of the addition in the current model of a realistic geometry (Wittig and Parsons, 2008; Graydon et al., 2011; Prokopiou and Drakakis, 2015) realistic, bursting single-channel kinetics for Ca channels (Magistretti et al., 2015) and intracellular Ca^2+^ stores (Castellano-Muñoz and Ricci, 2014) in modulating release dynamics. In particular, given that CICR and Ca current inactivation are able to introduce frequency-dependent distortions of the response, their role will be studied in depth.

### Conflict of interest statement

The authors declare that the research was conducted in the absence of any commercial or financial relationships that could be construed as a potential conflict of interest.
